# Towards a Rigorous Assessment of Systems Biology Models: The DREAM3 Challenges

**DOI:** 10.1371/journal.pone.0009202

**Published:** 2010-02-23

**Authors:** Robert J. Prill, Daniel Marbach, Julio Saez-Rodriguez, Peter K. Sorger, Leonidas G. Alexopoulos, Xiaowei Xue, Neil D. Clarke, Gregoire Altan-Bonnet, Gustavo Stolovitzky

**Affiliations:** 1 IBM T. J. Watson Research Center, Yorktown Heights, New York, United States of America; 2 Laboratory of Intelligent Systems, Ecole Polytechnique Fédérale de Lausanne, Lausanne, Switzerland; 3 Department of Systems Biology, Harvard Medical School, Boston, Massachusetts, United States of America; 4 Department of Biological Engineering, Massachusetts Institute of Technology, Cambridge, Massachusetts, United States of America; 5 Department of Mechanical Engineering, National Technical University of Athens, Athens, Greece; 6 Genome Institute of Singapore, Singapore, Singapore; 7 Program in Computational Biology and Immunology, Memorial Sloan-Kettering Cancer Center, New York, New York, United States of America; Center for Genomic Regulation, Spain

## Abstract

**Background:**

Systems biology has embraced computational modeling in response to the quantitative nature and increasing scale of contemporary data sets. The onslaught of data is accelerating as molecular profiling technology evolves. The Dialogue for Reverse Engineering Assessments and Methods (DREAM) is a community effort to catalyze discussion about the design, application, and assessment of systems biology models through annual reverse-engineering challenges.

**Methodology and Principal Findings:**

We describe our assessments of the four challenges associated with the third DREAM conference which came to be known as the DREAM3 challenges: signaling cascade identification, signaling response prediction, gene expression prediction, and the DREAM3 *in silico* network challenge. The challenges, based on anonymized data sets, tested participants in network inference and prediction of measurements. Forty teams submitted 413 predicted networks and measurement test sets. Overall, a handful of best-performer teams were identified, while a majority of teams made predictions that were equivalent to random. Counterintuitively, combining the predictions of multiple teams (including the weaker teams) can in some cases improve predictive power beyond that of any single method.

**Conclusions:**

DREAM provides valuable feedback to practitioners of systems biology modeling. Lessons learned from the predictions of the community provide much-needed context for interpreting claims of efficacy of algorithms described in the scientific literature.

## Introduction

Computational models of intracellular networks are a mainstay of systems biology. Researchers have used a variety of algorithms to deduce the structure of very different biological and artificial networks [1] and have evaluated their success using various metrics [2]–[8]. What is needed is a fair comparison of the strengths and weaknesses of the methods and a clear sense of the reliability of the network models they produce.

The Dialogue on Reverse Engineering Assessment and Methods (DREAM) project “takes the pulse” of the current state of the art in systems biology modeling [9], [10]. DREAM is organized around annual reverse-engineering challenges whereby teams download data sets from recent unpublished research, then attempt to recapitulate some withheld details of the data set. A challenge typically entails inferring the connectivity of the molecular networks underlying the measurements, predicting withheld measurements, or related reverse-engineering tasks. Assessments of the predictions are blind to the methods and identities of the participants.

The format of DREAM was inspired by the Critical Assessment of techniques for protein Structure Prediction (CASP) [11] whereby teams attempt to infer the three-dimensional structure of a protein that has recently been determined by X-ray crystallography but temporarily withheld from publication for the purpose of creating a challenge. Instead of protein structure prediction, DREAM is focused on network inference and related topics that are central to systems biology research. While no single test of an algorithm is a panacea for determining efficacy, we assert that the DREAM project fills a deep void in the validation of systems biology algorithms and models. The assessment provides valuable feedback for algorithm designers who can be lulled into a false sense of security based on their own internal benchmarks. Ultimately, DREAM and similar initiatives may demystify this important but opaque area of systems biology research so that the greater biological research community can have confidence in this work and build new experimental lines of research upon the inferences of algorithms.

### Evolution of the DREAM Challenges

At the conclusion of the second DREAM conference [10], a few voices of reason suggested that reverse-engineering challenges should not be solely focused on the network inference. As the argument goes, only that which can be measured should be predicted. Since knowledge of biological networks is actually a model in its own right, it may be counterproductive to evaluate networks for which no ground truth is known. We agree that the positivist viewpoint has merit both as a matter of philosophy and practicality. Some of the DREAM3 challenges reflect this attitude, which was a shift from previous challenges which were squarely focused on network inference.

Nevertheless, the systems biology community continues to assert—through funding opportunities, conference attendance, and the volume of publications—that network inference is a worthwhile scientific endeavor. Therefore, DREAM continues to provide a venue for vetting algorithms that are claimed to reverse-engineer networks from measurements. Despite the above mentioned criticisms, network inference challenges are a mainstay of DREAM. To contend with the criticism that no ground truth is known for molecular networks, the organizers must occasionally tradeoff realism for truth—generating *in silico* (i.e., simulated) data is one way that this problem is mitigated.

We describe the results of the DREAM3 challenges: signaling cascade identification, signaling response prediction, gene expression prediction, and *in silico* network inference. The fourth challenge was similar to the DREAM2 *in silico* network inference challenge [10] which enabled a cursory analysis of progress (or lack thereof) in the state of the art of network inference. The best-performer strategies in each challenge are described in detail in accompanying publications in this PLoS ONE Collection. Here, our focus is the characterization of the efficacy of the reverse-engineering community as a whole. The results are mixed: a handful of best-performer teams were identified, yet the performance of most teams was not all that different from random.

In the remainder of the Introduction we describe each of the four DREAM3 challenges. In Results and Discussion, we summarize the results of the prediction efforts of the community, identify best-performer teams, and analyze the impact of the community as a whole. Readers interested in a particular challenge can read the corresponding sub-sections without loss of continuity. Finally, we present the conclusions of this community-wide experiment in systems biology modeling.

## The DREAM3 Challenges

In this section we describe the challenges as they were presented to the participants. Additionally, we elaborate on the experimental methods at a level of detail that was not provided to the participants. We then go on to describe the basis of the assessments—“gold standard” test sets, scoring metrics, null models and *p*-values. The complete challenge descriptions and data are archived on the DREAM website [12].

### Signaling Cascade Identification

The signaling cascade identification challenge explored the extent to which signaling proteins are identifiable from flow cytometry data. Gregoire Altan-Bonnet of Memorial Sloan-Kettering Cancer Center generously donated the data set consisting of pairwise measurements of proteins that compose a signaling pathway in T cells [13]. The data producer, cell type, and protein identities were not disclosed to participants until the results of the challenge were released.

**Figure 1:**
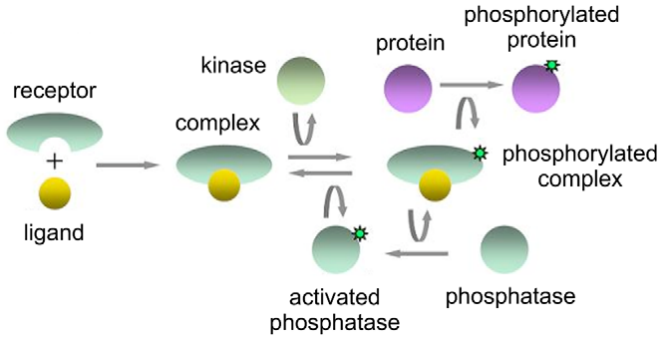
The objective of the signaling cascade identification challenge was to identify some of the molecular species in this diagram from single-cell flow cytometry measurements. The upstream binding of a ligand to a receptor and the downstream phosphorylation of a protein are illustrated.

Protein concentrations in a signaling network were measured in single cells by antibody staining and flow cytometry. Participants were provided a network diagram (Figure 1) and pairwise measurements of four signaling proteins (denoted x1, x2, x3, x4) obtained from single cells. The pairs of proteins (x1, x4), (x2, x4), and (x3, x4) were simultaneously measured in separate assays. The task was to identify each of the measured proteins (x1, x2, x3, x4) from among the seven molecular species (complex, phosphorylated complex, protein, phosphorylated protein, kinase, phosphatase, and activated phosphatase).

The experimental setup allowed for external control over the signaling network through the ligand that binds to the membrane-bound receptor. Two types of ligands, weak and strong (i.e., with different potency), in different concentrations, were used. Five concentrations of strong ligand (including none) and seven concentrations of weak ligand (including none) were applied to approximately 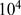 cells in separate experiments. In total, data from 36 experiments corresponding to the various combinations of quantified proteins, ligand type, and ligand concentration were provided. The biological motivation of the T cell experiment is discussed in [13].

#### Basis of assessment

Participants were instructed to identify each of each of the four measured proteins (x1, x2, x3, x4) as a molecular species (kinase, phosphatase, etc.). Each measurement could only be identified as a single molecular species, and each molecular species could be assigned to at most one measurement. For example, if measurement x1 was identified as the kinase then no other measurement could also be identified as the kinase. Submissions were scored by the probability that a random assignment table would result in as many correct identifications as achieved by the participant.

There are 840 possible assignment tables for seven molecular species and four measurements (i.e., 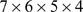). The probability of guessing the gold standard assignment table by chance is 1/840, which we denote 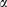. By enumerating the 840 tables and counting the number of correct (or incorrect) assignments in each table, we obtain the probability of correctly identifying four, three, two, one, or zero molecular species. It can be shown that the probability P() of making some number of correct identifications is exactly
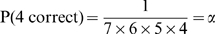


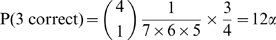


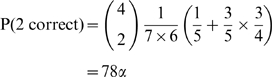


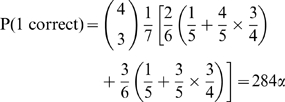


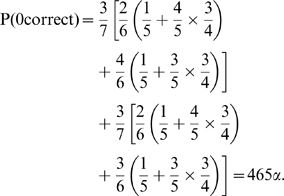
In addition to assigning a score to each team, we characterized the efficacy of the community as a whole. For example, what is the probability that five teams would correctly identify the same protein? To compute *p*-values for community-wide outcomes such as this we used the binomial distribution which is explained in Results.

### Signaling Response Prediction

The signaling response prediction challenge explored the extent to which the responses to perturbations of a signaling pathway can be predicted from a set of training data consisting of perturbations (environmental cues and signaling protein inhibitors) and their responses. Peter Sorger of Harvard Medical School generously donated the data for this challenge consisting of time-series measurements of a signaling network measured in human hepatocytes [14], [15]. The task entailed predicting some phosphoprotein and cytokine measurements that were withheld from the participants.

Approximately 10,000 fluorescence measurements proportional to the concentration of intracellular phosphorylated proteins and extracellular cytokines were acquired in normal human hepatocytes and the hepatocellular carcinoma cell line HepG2 using the using the Luminex (Austin, TX) 200 xMAP system. The data set consisted of measurements of 17 phosphoproteins at 0 minutes, 30 minutes, and 3 hrs following stimulation/perturbation of the two cell types. Additionally, 20 cytokines were quantified at 0 minutes, 3 hours, and 24 hours following stimulation/perturbation of the two cell types. Data were processed and visualized using the open-access MATLAB-based software, DataRail [16]. The cell types and protein identities were disclosed so that participants could draw upon the existing signal transduction literature.

**Figure 2:**
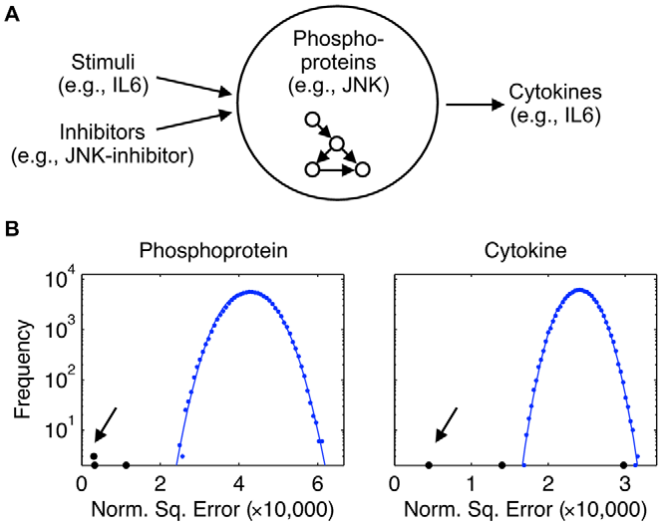
The objective of the signaling response prediction challenge was to predict the concentrations of phosphoproteins and cytokines in response to combinatorial perturbations the environmental cues (stimuli) and perturbations of the signaling network (inhibtors). (**a**) A compendium of phosphoprotein and cytokine measurements was provided as a training set. (**b**) Histograms (log scale) of the scoring metric (normalized squared error) for 100,000 random predictions were approximately Gaussian (fitted blue points). Significance of the predictions of the teams (black points) was assessed with respect to the empirical probability densities embodied by these histograms. Scores of the best-performer teams are denoted with arrows.

In each experiment, a combination of a single chemical stimulus and a single chemical perturbation to the signaling network were simultaneously applied, and measurements of either the signaling network proteins or cytokines were taken (Figure 2A). Seven stimuli were investigated: INF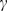, TNF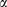, IL1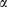, IL6, IGF-I, TGF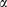, and LPS (Table 1). Also, seven chemical inhibitors of specific signaling proteins were investigated, which selectively inhibited the activities of MEK12, p38, PI3K, IKK, mTOR, GSK3, or JNK. All pairs of stimulus/inhibitor combinations (a total of 64) were applied to both cell types and measurements of fluorescence for individual proteins or cytokines were taken at the indicated time points. Fluorescence was reported in arbitrary units from 0 to 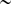
29000. The upper limit corresponded to saturation of the detector. Signal intensity below 300 was considered noise. Fluorescence intensity was approximately linear with concentration in the mid-dynamic range of the detector.

**Table 1:** The signaling response prediction challenge solicited predictions of the concentrations of 17 phosphoproteins and 20 cytokines.

Phosphoproteins (17)	Inhibitors (7)	Cytokines (20)	Stimuli (7)
Akt		IL1b	
ERK1/2		IL4	
GSK-3alpha/beta		IL6	IL6
IkappaB-alpha		IL8	
JNK	JNK-i	IL10	
p38 MAPK	p38-i	IL15	
p70 S6 kinase		GCSF	
p90RSK		GMCSF	
STAT3		IP10	
c-Jun		MCP1	
CREB		MIP1a	
Histone H3		MIP1b	
HSP27		PDGFbb	
IRS-1		RANTES	
MEK1		VEGF	
p53		GROa	
STAT6		ICAM1	
	MEK12-i	MIF	
	PI3K-i	MIG	
	IKK-i	SDF1a	
	mTOR-i		INFg
	GSK3-i		TNFa
			IL1a
			IGF-I
			TGFa
			LPS

The data set underlying this challenge consisted of phosphoprotein and cytokine concentrations in response to 49 combinatoric perturbations of seven protein-specific inhibitors and seven stimuli.

The challenge was organized in two parts that were evaluated separately: the phosphoprotein subchallenge and the cytokine subchallenge. The complete data set (training and test) in the signaling response prediction challenge was composed of fluorescence measurements of phosphoproteins and cytokines in cells exposed to pairwise combinations of eight stimuli and eight signaling-network-protein inhibitors, for a total of 64 stimulus/inhibitor combinations (including zero concentrations). Fifty-seven of the combinations composed the training set, and seven combinations composed the test set. The phosphoprotein subchallenge solicited predictions for 17 phosphoproteins, in two cell types (normal, carcinoma), at two time points, under seven combinatoric stimulus/inhibitor perturbations for a total of 476 predictions. Likewise, the cytokine subchallenge solicited predictions for 20 cytokines for a total of 560 predictions. The biological motivation for the hepatocyte experiment is described in [14], [15].

#### Basis of assessment

Assessment of the predicted measurements was based on a single metric, the normalized squared error over the set of predictions in each subchallenge,
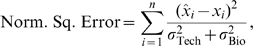
(1)where 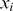 is the 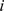
th measurement, 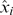 is the 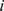
th prediction, 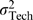 is the technical variance, and 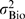 is the biological variance. The variances were parametrized as follows: 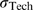 = 300 (minimum sensitivity of the detector for antibody-based detection assays) and 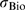 = 0.8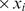 (product of the coefficient of variation and the measurement). Note that the squared prediction error is normalized by an estimate of the measurement variance, a sum of the biological variance and the technical variance. A probability distribution for this metric was estimated by simulation of a null model.

The null model was based on a naive approach to solving the challenge. Essentially, participants were provided a spreadsheet of measurements with some entries missing. The columns of the spreadsheet corresponded to the phosphoproteins or cytokines (depending on the subchallenge); rows corresponded to various perturbations of stimuli and inhibitors. We randomly “filled-in” the spreadsheet by choosing values for the missing entries, with replacement, from the corresponding column. Since each protein or cytokine had a characteristic dynamic range, this procedure ensured that the random predictions were drawn from the appropriate order of magnitude of fluorescence. This procedure was performed 100,000 times. Parametric curves were fit to the histograms (Figure 2B) to extrapolate the probability density beyond the range of the histogram (i.e., to compute *p*-values for teams that did far better or worse than this null model). The procedure for curve-fitting was described previously [10]. Briefly, an approximation of the empirical probability density was given by stretched exponentials with different parameters to the right and left of the mode of the distribution, with functional form
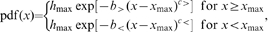
(2)where 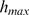 is the maximum height of the histogram, 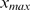 is the position of 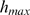, and 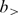, 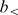, 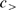, and 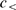 are fitted parameters.

### Gene Expression Prediction

The gene expression prediction challenge explored the extent to which time-dependent gene expression measurements can be predicted in a mutant strain of *S. cerevisiae* (budding yeast) given complete expression data for the wild type strain and two related mutant strains. Experimental perturbations involving histidine biosynthesis provided a context for the challenge. Neil Clarke of the Genome Institute of Singapore generously donated unpublished gene expression measurements for this challenge.

The yeast transcription factors GAT1, GCN4, and LEU3 regulate genes involved in nitrogen and/or amino acid metabolism. They were disrupted in three mutant strains denoted 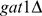, 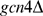, and 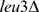. These genes are considered nonessential since the deletion strains are viable. Expression levels were assayed separately in the three mutant strains and in the wild type strain at times 0, 10, 20, 30, 45, 60, 90 and 120 minutes following the addition of 3-aminotriazole (3AT) as described [17]. 3AT inhibits an enzyme in the histidine biosynthesis pathway and, in the appropriate media (used in these experiments), has the effect of starving the cells for this essential amino acid.

Expression measurements were obtained using DNA microarrays (Affymetrix YGS98 GeneChip). Two biological replicates (i.e., independent cultures) and an additional technical replicate (i.e., independent labeling and hybridization of the same culture) were performed. Measurements were normalized using the RMA algorithm [18] within the commercial software package, GeneSpring. Values were median normalized within arrays prior to the calculation of fold-change. The mean hybridization value for each probe set was obtained from the three replicates and was normalized to the mean value for the probe set in the wild-type samples at time zero. Values were provided as the log (base two) of the ratio of the indicated experimental condition (i.e., strain and time point) relative to the wild type strain at time zero.

The fifty genes composing the test set were selected by subjective criteria, but with an eye towards enriching for genes that are significantly regulated in at least one strain, or are bound by one or more of the transcription factors according to ChIP-chip data, or are relatively strongly predicted to be bound based on a PWM-based promoter occupancy calculation [19]. Thus, the expression profiles for these genes tend to be somewhat more explicable than would be the case for randomly selected genes. Nevertheless, it was trivial to find genes for which an explanation for the expression profiles was not obvious, and there are many such genes among the fifty prediction targets.

A quantitative prediction of gene expression changes is far beyond state of the art at this time, so participants were asked to predict the relative expression levels for 50 genes at the eight time points in the 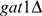 strain. Participants were provided complete expression data for the other strains, as well measurements for the genes that were not part of the set of 50 challenge genes in 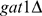. Predictions for each time point were submitted as a ranked list with values from 1 to 50 sorted from most induced to most repressed compared to the wild type expression at time zero.

#### Basis of assessment

Participants submitted a spreadsheet of 50 rows (genes) by eight columns (time points). Submissions were scored using Spearman's rank correlation coefficient between the predicted and measured gene expression at each of the eight time points. The same statistic was also computed with respect to each gene across all time points. Thus, we evaluated predictions using two different tests of similarity to the gold standard which we call the time-profiles and gene-profiles, respectively.

For each column of the predicted matrix of relative expression, we obtained a correlation coefficient and its corresponding *p*-value under the null hypothesis that the ranks are randomly distributed. From the column *p*-values we arrived at a single summary *p*-value for all eight time points using the geometric mean of individual *p*-values (i.e., 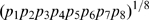). The same procedure was performed on the row *p*-values to arrive at a summary *p*-value for the 50 genes. Finally, the score used to assess best-performers was computed from the two summary *p*-values
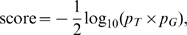
(3)where 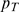 is the overall *p*-value for the time-profiles (columns) and 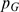 is the overall *p*-value for the gene-profiles (rows). The higher the score, the more significant the prediction.

### *In Silico* Network Inference

The *in silico* network inference challenge explored the extent to which gene networks of various sizes and connection densities can be inferred from simulated data. Daniel Marbach of Ecole Polytechnique Fédérale de Lausanne extracted the challenge networks as subgraphs of the currently accepted *E. coli* and *S. cerevisiae* gene regulation networks [8] and imbued the networks with dynamics using a thermodynamic model of gene expression. The *in silico* “measurements” were generated by continuous differential equations which were deemed reasonable approximations of gene expression regulatory functions. To these values was added a small amount of Gaussian noise to simulate measurement error.

The simulated data was meant to mimic three typical types of experiments: (1) time courses of a wild type strain following an environmental perturbation (i.e., trajectories); (2) knock-down of a gene by deletion of one copy in a diploid organism (i.e., heterozygous mutants); (3) knock-out of a gene by deletion of both copies in a diploid organism (i.e., homozygous null mutants). Technically, a haploid organism such as *E. coli* can not be a heterozygote, but since this data only exists *in silico* we did not see harm in the use of this term. A trajectory of the wild-type response to an environmental perturbation was simulated by a random initialization of the simulation. A heterozygous knock-down mutant was simulated by halving the wild type concentration of the gene. A homozygous knock-out mutant was simulated by flooring the wild type concentration of the gene to zero.

The challenge was organized into three parts: the 10-node subchallenge, the 50-node subchallenge, and the 100-node subchallenge. Within each sub-challenge, participants were required to predict five networks, denoted Ecoli1, Ecoli2, Yeast1, Yeast2, Yeast3. Completion of a subchallenge required submission of predictions for all five of the networks in the subchallenge. Participants were encouraged, but not required, to perform all three subchallenges on networks of various sizes. Some of the gross topological properties of the fifteen gold standard networks are illustrated in Table 2.

**Table 2:** Statistical properties of the gold standard networks in the *in silico* network inference challenge.

Sub-challenge	Network	Nodes	Edges	Regulators
***In Silico*** ** Size 10**	Ecoli1	10	11	5
	Ecoli2	10	15	3
	Yeast1	10	10	7
	Yeast2	10	25	8
	Yeast3	10	22	9
***In Silico*** ** Size 50**	Ecoli1	50	62	13
	Ecoli2	50	82	11
	Yeast1	50	77	26
	Yeast2	50	160	37
	Yeast3	50	173	35
***In Silico*** ** Size 100**	Ecoli1	100	125	26
	Ecoli2	100	119	19
	Yeast1	100	166	60
	Yeast2	100	389	71
	Yeast3	100	551	81

In each of the three sub-challenges the number of nodes was held constant but the number of edges and regulator nodes was not. There were five gold standard networks in each of the three sub-challenges (which were treated as three separate contests).

Complete steady state expression information was provided for the wild type and mutant strains. In other words, in the 10-node subchallenge, all ten genes were knocked-down and knocked-out, one at a time, while the remaining nine measurements were provided. Various numbers of trajectories from random initializations were provided depending on the subchallenge. Four, 23, and 46 trajectories were provided for the 10-node, 50-node, and 100-node subchallenges, respectively.

Participants were asked to predict the directed, unsigned networks from *in silico* gene expression data sets. A network prediction was submitted in the form of a ranked list of potential network edges ordered from most reliable to least reliable. In other words, the edges at the top of the list were believed to be present in the network and the edges at the bottom of the list were believed to be absent from the network. This submission format was chosen because it does not require the researcher to impose a particular threshold for calling an edge present or absent. Also, it can be scored without imposition of a specific threshold. An example of the file format of a network prediction is illustrated in Table 3.

**Table 3:** Format of a predicted network in the *in silico* network inference challenge.

Source Node	Target Node	Confidence	Scoring Cutoff (k)
G85	G1	1.00	1
G85	G10	0.99	2
G10	G85	0.73	3
G99	G52	0.44	4
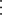	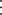	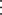	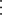
G10	G3	0.01	N(N-1)

Predicted edges were to be ranked from most confidence to least confidence that the edge is present in the network. A directed edge is denoted by a source and target node and an arbitrary (non-increasing) score between one (most confidence) to zero (least confidence). Thus, edges that are predicted to exist in the network should be at the top of the list and those predicted not to exist in the network should be at the bottom of the list. To evaluate the predicted network, two metrics—area under the ROC curve and area under the precision-recall curve—were computed by scanning all possible decision boundaries (i.e., k = 1, k = 2, etc.) up to the maximum number of possible directed edges (excluding self-edges).

#### Basis of assessment

From the ranked edge-list (Table 3), a particular concrete network with 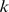 edges is obtained by designating the first 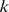 edges present and the remaining edges absent. Then, 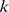 is a parameter that controls the number of edges in a predicted network. Various performance metrics were computed as 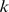 was varied from 1 to 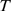, the total number of possible directed edges, where 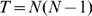 and 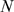 is the number of nodes in the network.

Two parametric curves, the precision-recall (P-R) curve and the the receiver operating characteristic (ROC) curve, were traced by scanning 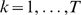. *Recall* is a measure of completeness,
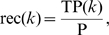
where TP(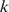) is the number of true positives at threshold 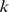, and P is the number of positives (i.e., gold standard edges). *Precision* is a measure of fidelity,
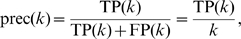
where FP(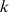) is the number of false positives at threshold 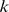. Note that the sum of TP(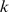) and FP(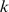) is 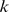. The precision-recall curve graphically explores the tradeoff between these complementary metrics as the parameter 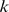 is varied. The area under the precision-recall curve (AUPR) is a single number that summarizes the precision-recall tradeoff. Similarly, the receiver operating characteristic (ROC) curve graphically explores the tradeoff between the *true positive rate* (TPR) and the *false positive rate* (FPR). TPR(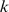) is the fraction of positives that are correctly predicted at threshold 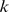,
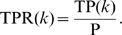
(Note that TPR is equivalent to recall.) FPR(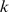) is the fraction of negatives that are incorrectly predicted at threshold 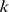,
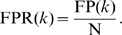

*Negative* denotes the absence of an edge in the gold standard network. The area under the ROC curve (AUROC, also denoted AUC in the literature) is a single number that summarizes the tradeoff between TPR(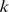) and FPR(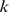) as the parameter 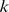 is varied. Using both the AUPR and the AUROC metrics, we gain a fuller characterization of the prediction than using either alone. For example, the P-R curve indicates whether the first few edge predictions at the top of the prediction list are correct. The ROC curve does not provide this information.

A technical point is the issue of how to score a truncated prediction list, where fewer than the total number of possible edges are submitted. A methodology is in place from the previous DREAM assessment [10]. If a prediction list does not contain a complete ordering of all possible 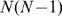 edges, we “add” the missing edges in random order at the end of the list. The addition takes place in an analytical way.

A team's score for a subchallenge depended on quite a few calculations. Each of the five network predictions (Ecoli1, Ecoli2, Yeast1, Yeast2, Yeast3) were evaluated by AUPR and AUROC. *P*-values for these assessments were obtained from the empirical distributions described above. The five AUPR *p*-values were condensed to an overall AUPR *p*-value using the geometric mean of individual *p*-values (i.e., 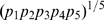). The same procedure was performed on the five AUROC *p*-values to arrive at an overall AUROC *p*-value. Finally, the score for the team was computed as
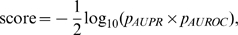
(4)where 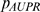 and 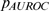 are the overall *p*-values for AUPR and AUROC, respectively. The higher the score, the more significant the network prediction.

## Results and Discussion

The DREAM3 challenges were posted on the DREAM website on June 15, 2008. Submissions in response to the challenges were accepted on September 15, 2008. Forty teams submitted 413 predicted networks and test set predictions in the various challenges. The anonymous results were posted on the DREAM website [12] on October 15, 2008.

In this section, we describe our assessment of the predictions supplied by the community. Our dual goals are to identify the best-performers in each challenge and to characterize the efficacy of the community as a whole. We highlight the best-performer strategies and comment on some of the sub-optimal strategies. Where possible, we attempt to leverage the community intelligence by combining the predictions of multiple teams into a consensus prediction.

Best-performers in each challenge were identified by statistical significance with respect to a null model combined with a clear delineation from the rest of the participating teams (e.g., an order of magnitude lower *p*-value compared to the next best team). Occasionally, this criterion identified multiple best-performers in a challenge.

### Signaling Cascade Identification

Seven teams submitted predictions for the signaling cascade identification challenge as described in the Introduction. Submissions were scored based on the probability that a random solution to the challenge would achieve at least as many correct protein identifications as the submitted solution.

Five of seven teams identified two of the four proteins correctly (though not the same pair) (Table 4). One team identified only one protein correctly and one team did not identify any correctly. The *p*-value for a team identifying two or more proteins correctly is 0.11, as described in the Introduction. On the basis of this *p*-value, this challenge did not have a best-performer. However, in the days following the conference, follow-up questions from some of the participants to the data provider revealed a misrepresentation in how the challenge was posed, which probably negatively impacted the teams' performances. The source of the confusion is describe below.

**Table 4:** Results of the signaling cascade identification challenge.

	No. Correct	
Team	Identifications	*p*-value
Team 315	2	0.11
Team 283	2	0.11
Team 106	2	0.11
Team 281	2	0.11
Team 181	2	0.11
Team 110	1	0.45
Team 286	0	1.00

Two correct identifications is not a significant performance so no team was named the best-performer.

Despite that no individual team gained much traction in solving this challenge, the community as a whole seemed to possess intelligence. For example, five of seven teams correctly identified two proteins (though not the same pair). While such a performance is not significant on an individual basis, the event of five teams correctly identifying two proteins is unlikely to occur by chance. Under the binomial distribution, assuming independent teams, the probability of five or more teams correctly identify two or more proteins is 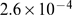.

**Figure 3:**
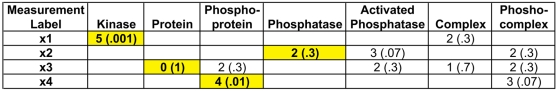
Overlay of the assignment tables from the seven teams in the signaling cascade identification challenge. The number of teams making each assignment and the *p*-value is indicated. The *p*-value expresses the probability of a such a concentration of random guesses in the same table entry. Highlighted entries are correct. Five teams correctly identified species x1 as the kinase, a significant event for the community despite that no team had a significant individual performance.

Summing over the predictions of all the teams we obtain Figure 3. For example, five of seven teams correctly identified x1 as the kinase. The probability that five or more teams would pick the same table entry is 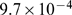. Similarly, the probability of three or more teams identifying the same pair of proteins (e.g., kinase, phosphoprotein) is 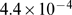.

The assumption of independence is implicit in the null hypothesis underlying these *p*-values. Rejection of the null hypothesis on the basis of a small *p*-value indicates that there is a correlation between the teams. This correlation can be interpreted as a shared success within the community. In other words, the community exhibits some intelligence not evidenced in the predictions of the individual teams. Based on this assessment of the community as a whole, we conclude that some structural features of the signaling cascade were indeed identified from flow cytometry data.

The community assessment suggests that a mixture of methods may be an advantageous strategy for identifying signaling proteins from flow-cytometry data. A simple strategy for generating a consensus prediction is illustrated by Figure 3 in which the total number of predictions made by the community for each possible assignment are indicated along with the corresponding *p*-values indicating the probability of such a concentration of predictions in a single table entry. The the kinase and phosphorylated protein are the only identifications (individually) significant at 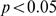. This analysis also reveals clustering of incorrect predictions—the phosphatase was most often confused with the activated phosphatase, and the phosphorylated protein was most often confused with the phosphorylated ligand-receptor complex—but these misidentifications were not significant.

#### Mea culpa: a poorly posed challenge

There are three conjugate pairs of species in the signaling pathway: complex/phospho-complex, protein/phosho-protein, and phosphatase/activated phosphatase. The challenge description led participants to believe that each measured species (x1,…, x4) may match one of the six individual species. In fact, measurement x3 corresponded to total protein (inactive and active forms). Likewise, measurement x2 corresponded to total phosphatase (inactive and active forms). It would be highly unusual for an antibody to target one epitope of a protein to the exclusion of a phosphorylated epitope. That is, it would be difficult but not impossible to raise an antibody that reacted with only the unphosphorylated version of a protein. This serious flaw in the design of the challenge did not come to light until after the scoring was complete.

The simultaneous identification of the upstream kinase and the downstream phosphorylated protein (Figure 3) can be explained in light of the confusion surrounding precisely what the measurements entailed. The measurements corresponding to the kinase and phosphoprotein were accurately portrayed in the challenge description whereas the total protein and total phosphatase were not.

### Signaling Response Prediction

Four teams participated in the signaling response prediction challenge. The phosphoprotein subchallenge received three submissions, as did the cytokine subchallenge. As described in the Introduction, the task was to predict measurements of proteins and/or cytokines, in normal and cancerous cells, for combinatoric perturbations of stimuli and inhibitors of a signaling pathway. Submissions were scored by a metric based on the sum of the squared prediction errors (Figure 2B).

In the phosphoprotein subchallenge two teams achieved a *p*-value orders of magnitude lower than the remaining other submission (Table 5). In the cytokine subchallenge one team had a substantially smaller total prediction error than the next best team. On this basis, the best-performers were:

- Genome Singapore (phosphoprotein and cytokine subchallenges): Guillaume Bourque and Neil Clarke of the Genome Institute of Singapore, Singapore
- Vital SIB (phosphoprotein subchallenge): Nicolas Guex, Eugenia Migliavacca, and Ioannis Xenarios of the Swiss Institute of Bionformatics, Switzerland

**Table 5:** Results of the signaling response prediction challenge.

Subchallenge	Team	Norm. Sq. Error	*p*-value
Phosphoprotein	Vital SIB	3102	2 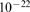
	GenomeSingapore	3310	4 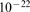
	Team 302	11329	7 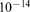
Cytokine	GenomeSingapore	4462	8 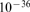
	Team 302	13995	4 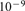
	Team 126	29795	1

There are two main types of strategies that could have been employed in this challenge: to explicitly model the underlying signaling network, or to model the data statistically. Both of the best-performers took a statistical approach. Vital SIB approached it as a missing data problem and used multiple imputation to predict the missing data. This involved learning model parameters by cross-validation, followed by prediction of the missing data [20]. Genome Singapore identified the nearest-neighbors of missing measurements based on similarity of the measurement profiles [21]. To predict the measurements for an unobserved stimulus or inhibitor, they took into consideration the values observed for the nearest neighbor. Neither team utilized external data sources, nor did they evoke the concept of a biological signaling network.

Surprisingly, one team in the cytokine subchallenge had a significantly larger total error than random. We investigated this strange outcome further. This team systematically under-predicted the medium and large intensity measurements (data not shown). This kind of systematic error was heavily penalized by the scoring metric. Nevertheless, the best-performer would have remained the same had linear correlation been used as the metric. Due to the low participation level from the community, we did not perform a community-wide analysis.

### Gene Expression Prediction

Nine teams participated in the gene expression prediction challenge as described in the Introduction. The task was to predict the expression of 50 genes in the 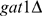 strain of *S. cerevisiae* at eight time points. Participants submitted a spreadsheet of 50 rows (genes) by eight columns (time points). At each time point, the participant ranked the genes from most induced to most repressed compared to the wild type values at time zero. Predictions were assessed by Spearman's correlation coefficient and its corresponding *p*-value under the null hypothesis that the ranks are uniformly distributed.

The *p*-values (based on Spearman correlation coefficient) computed over the set of 50 test genes at each of the eight time-points are reported in Table 6. Some trends are readily identifiable. Across the community, the least significant predictions were those at time zero. Relatively more significant predictions were made at 10, 20, 45, and 60 minutes, and comparatively less significant predictions were made at 30 and 90 minutes. This analysis identified the teams that predicted well (over the 50 test genes) at each time point. We computed a summary statistic for each team using the geometric mean of the eight *p*-values for the individual time points.

**Table 6:** Time-profile *p*-values of the gene expression prediction challenge.

				Minutes					Summary
	0	10	20	30	45	60	90	120	Time-profile
Team	*p*-value	*p*-value	*p*-value	*p*-value	*p*-value	*p*-value	*p*-value	*p*-value	*p*-value
Gustafsson-Hornquist	2 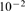	1 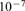	3 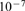	1 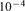	1 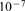	1 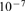	7 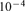	3 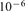	6.5 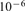
Dream Team 2008	3 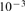	4 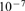	3 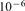	5 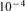	1 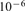	1 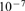	2 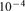	6 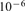	1.1 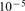
Team 263	7 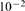	1 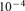	7 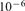	4 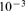	2 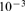	2 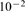	8 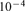	2 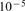	7.5 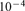
Team 297	8 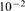	1 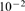	7 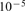	4 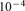	1 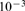	4 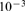	4 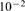	3 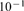	5.6 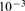
Team 126	1 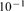	5 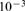	8 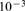	2 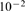	8 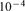	1 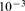	9 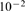	8 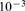	9.0 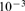
Team 273	3 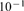	1 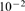	4 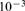	1	1 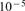	6 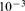	9 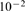	2 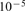	6.1 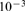
Team 186	1 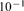	2 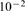	7 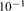	7 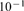	2 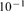	9 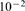	2 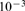	1 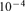	3.9 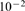
Team 190	3 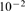	1 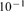	6 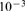	2 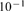	6 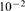	3 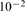	2 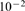	7 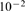	4.2 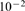
Team 193	1 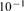	1	1	8 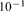	1	1	1	1	7.4 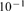

*P*-values at each time-point and a summary *p*-value (geometric mean) are indicated.

In the above analysis, each of the eight time points was analyzed as a 50-dimensional vector. An alternative viewpoint is to consider each of the 50 genes as an eight-dimensional vector. We also performed this analysis using Spearman's correlation coefficient computed for each gene. We computed a summary statistic for each team using the geometric mean of the 50 *p*-values for the individual genes (not shown). Correlation coefficients and *p*-values for the gene-profiles are published on the DREAM website [12].

Summary statistics from the time-profile analysis and the gene-profile analysis are reported in Table 7. Weaker significance of gene-profile *p*-values compared to time-profile *p*-values may be due to the fact that the former are eight-dimensional vectors while the latter are 50-dimensional vectors. Best-performers were identified by an overall score based on the time-profile and gene-profile summary *p*-values. A difference of one in the overall score corresponds to an order of magnitude difference in the *p*-value. Two teams performed more than an order of magnitude better than the nearest competitor at 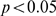.

- Gustafsson-Hornquist : Mika Gustafsson and Michael Hornquist of Linköping University, Sweden
- Dream Team 2008 : Jianhua Ruan of the University of Texas at San Antonio, USA

**Table 7:** Results of the gene expression prediction challenge.

	Time-profile	Gene-profile	Overall
Team	*p*-value	*p*-value	Score
Gustafsson-Hornquist	7 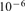	5 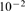	3.3
Dream Team 2008	1 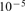	4 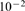	3.2
Team 263	8 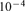	3 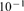	1.8
Team 297	6 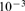	8 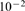	1.7
Team 126	9 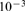	1 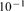	1.5
Team 273	6 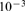	4 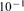	1.3
Team 186	4 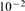	3 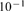	1.0
Team 190	4 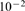	4 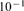	0.9
Team 193	7 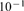	5 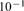	0.2

**Figure 4:**
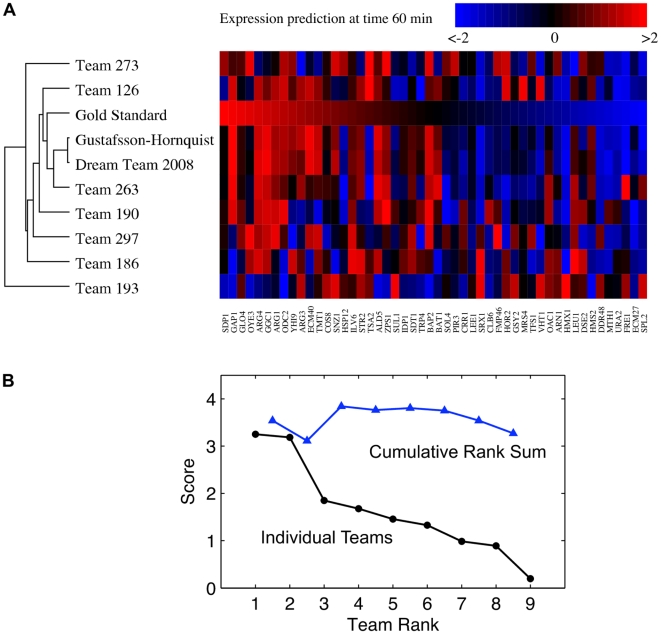
The objective of the gene expression prediction challenge was to predict temporal expression of 50 genes that were withheld from a training set consisting of 9285 genes. (**a**) Clustered heatmaps of the predicted genes (columns) reveal that two best-performer teams predicted substantially similar gene expression values, though different methods were employed. Results for the 60 minute time-point are shown. (**b**) The benefits of combining the predictions of multiple teams into a consensus prediction are illustrated by the rank sum prediction (triangles). Some rank sum predictions score higher than the best-performer, depending on the teams that are included. The highest score is achieved by a combination of the predictions of the best four teams.

We used hierarchically clustered heat maps to visualize the teams' predictions (gene ranks from 1 to 50) relative to the gold standard (Figure 4A). The two best-performers were more similar to each other than either was to the gold standard. The Spearman correlation coefficient between Gustafsson-Hornquist and Dream Team 2008 is 0.96, while the correlation between either team and the Gold Standard is 0.67. One could reasonably presume that substantially similar methods were employed by both teams. That turns out not the be the case.

Team Gustafsson-Hornquist used a weighted least squares approach in which the prediction for each gene was a weighted sum of the values of the other genes [22]. The particular linear model they employed is called an elastic net, which is a hybrid of the lasso and ridge regression [23]. They incorporated additional data into their model, taking advantage of public yeast expression profiles and ChIP-chip data. The additional expression profiles provided more training examples from which to estimate pairwise correlations between genes. The physical binding data (ChIP-chip) was integrated into the linear model by weighting each gene's contribution to a prediction based on the number of common transcription factors the pair of genes shared.

Dream Team 2008 did not use any additional data beyond what was provided in the challenge. Rather, they employed a 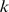-nearest neighbor (KNN) approach to predict the expression of a gene based on the expression of other genes in the same strain at the same time point [24]. The Euclidean distance between all pairs of genes was determined from the strains for which complete expression profiles were provided. The predicted value of a gene was the mean expression of the 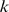-nearest-neighbors. The parameter 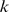 was chosen by cross-validation; 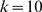 was used for prediction.

Does the community possess an intelligence that trumps the efforts of any single team? To answer this question we created a consensus prediction by summing the predictions of multiple teams, then re-ranking. The results of this analysis are shown in Figure 4B which traces the overall score of the consensus prediction as lower-significance teams are included. The first consensus prediction includes the best and second-best teams. The next consensus prediction includes the top three teams, and so on.

The consensus prediction of the top four teams had a higher score than the best-performer, which is counter-intuitive since the third and fourth place teams individually scored much lower than the best-performer (Figure 4B). Furthermore, the inclusion of all teams in the consensus prediction scored about the same as the best-performer. This result suggests that, given the output of a collection of algorithms, combining multiple result sets into a consensus prediction is an effective strategy for improving the results.

We assigned a difficulty level to each gene based on the accuracy of the community. For each gene, we computed the geometric mean of the gene-profile *p*-values over the nine teams, which we interpreted as the difficulty level of each gene. The five best-predicted genes were: *arg4*, *ggc1*, *tmt1*, *arg1*, and *arg3*. The five worst-predicted genes were: *srx1*, *lee1*, *sol4*, *glo4*, and *bap2*. The relative difficulty of prediction of a gene was weakly correlated with the absolute expression level of that gene at 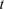 = 0, but many of the 50 genes defied a clear trend. The five best-predicted genes had an average expression of 42.7 (arbitrary units, log scale) at t = 0, whereas the five worst-predicted genes had an average expression of 3.7. It is known that low intensity signals are more difficult to characterize with respect to the noise. It is likely that the absolute intensity of the genes played a role in the relative difficulty of predicting their expression values.

### *In Silico* Network Inference

Twenty-nine teams participated in the *in silico* network inference challenge as described in the Introduction, the greatest level of participation by far of the four DREAM3 challenges. The task was to infer the underlying gene regulation networks from *in silico* measurements of environmental perturbations (dynamic trajectories), gene knock-downs (heterozygous mutants), and gene knock-outs (homozygous null-mutants). Participants predicted directed, unsigned networks as a ranked list of potential edges in order of the confidence that the edge is present in the gold standard network. Predictions for 15 different networks of various “real-world” inspired topologies were solicited, grouped into three separate subchallenges: the 10-node, 50-node, and 100-node subchallenges. The three subchallenges were evaluated separately.

**Figure 5:**
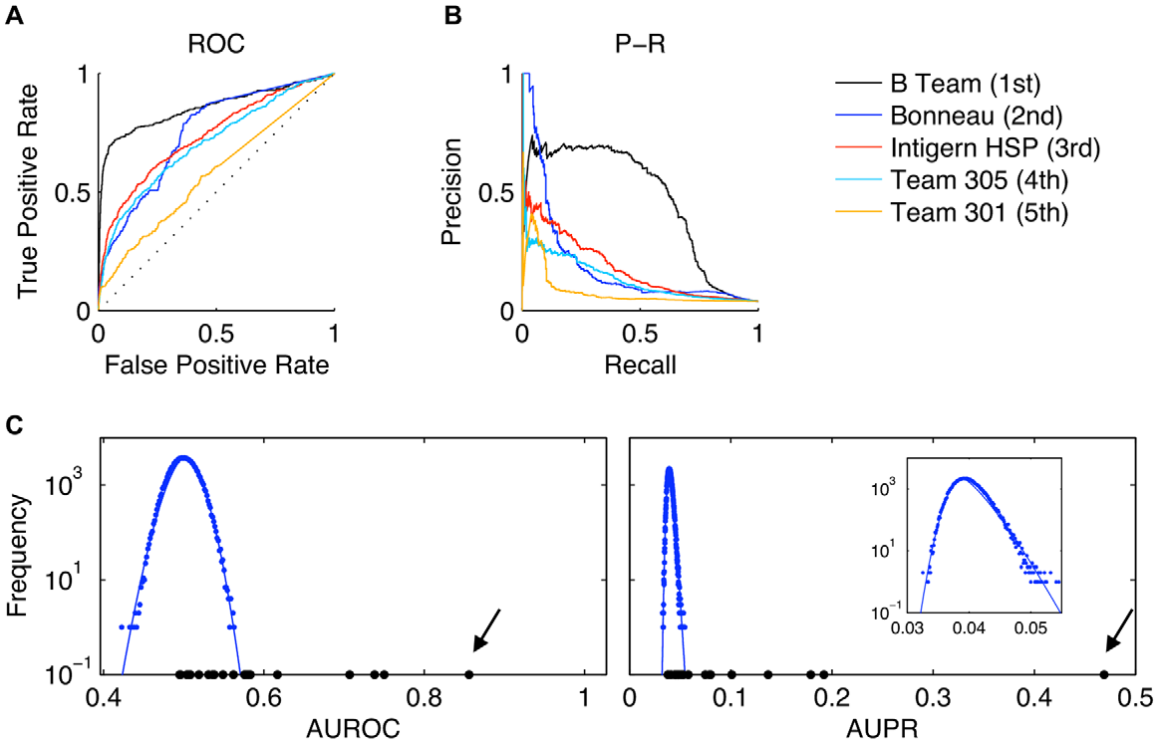
The objective of the *in silico* network inference challenge was to infer networks of various sizes (10, 50, and 100 nodes) from steady-state and time-series “measurements” of simulated gene regulation networks. Predicted networks were evaluated on the basis of two scoring metrics, (**a**) area under the ROC curve and (**b**) area under the precision-recall curve. ROC and precision-recall curves of the five best teams in the 100-node sub-challenge. (**a**) Dotted diagonal line is the expected value of a random prediction. (**b**) Note that the best and second-best performers have different precision-recall characteristics. (**c**) Histograms (log scale) of the AUROC scoring metric for 100,000 random predictions was approximately Gaussian (fitted blue points) whereas the histogram of the AUPR metric was not (inset). Significance of the predictions of the teams (black points) was assessed with respect to the empirical probability densities embodied by these histograms. Scores of the best-performer team are denoted with arrows. All plots are analyses of the gold standard network called *InSilico_Size100_Yeast2*.

Each predicted network was evaluated using two metrics, the area under the ROC curve (AUROC) and the area under the precision-recall curve (AUPR). To provide some context for these metrics we demonstrate the ROC and P-R curves for the five best teams in the 100-node subchallenge (Figure 5A, 5B). These complementary assessments enable valuable insights about the performance of the various teams.

Based on the P-R curve, we observe that the best-performer in this subchallenge actually had low precision at the top of the prediction list (i.e., the first few edge predictions were false positives), but subsequently maintained a high precision (approximately 0.7) to considerable depth in the prediction list. By contrast, the second-place team had perfect precision for the first few predictions, but precision then plummeted. In another example of the complementary nature of the two assessments, consider the fifth-place team. On the basis of the ROC, the fifth place team is scarcely better than random (diagonal dotted line) however, on the basis of the P-R curve, it is clear that the fifth place team achieved better precision than random at the top of edge list. The two types of curves are non-redundant and enable a fuller characterization of prediction performance than either alone.

ROC and P-R curves like those shown in Figure 5 were summarized using the area under the curve. The details of the calculation of the area under the ROC curve and the area under the P-R curve are described at length in [10]. Probability densities for AUPR and AUROC were estimated by simulation of 100,000 random prediction lists. Curves were fit to the histograms using Equation 2 so that the probability densities could be extrapolated beyond the ranges of the histograms in order to compute *p*-values for teams that predicted much better or worse than the null model. Figure 5C demonstrates the teams' scores in the reconstruction of the gold standard network called *InSilico_Size100_Yeast2*. The best-performer made an exceedingly significant network prediction (identified by an arrow) whereas many of the teams predicted equivalently to random.

Best-performers in each subchallenge were identified by an overall score that summarized the statistical significance of the five network reconstructions composing the subchallenge (Ecoli1, Ecoli2, Yeast1, Yeast2, Yeast3). The AUROC *p*-values for the 100-node subchallenge are indicated in Table 8. The complete set of tables for the other subchallenges are available on the DREAM website [12]. A summary *p*-value for AUROC was computed as the geometric mean of the five *p*-values. Likewise, a summary *p*-value for AUPR was computed (not shown). Finally, the overall score for a team was computed from the two summary *p*-values according to Equation 4 (Table 9). A difference of one in the score corresponds to an order of magnitude difference in *p*-value —the higher the score, the more significant the prediction. On the basis of the overall score, the same team was the best-performer in the 10-node, 50-node, and 100-node subchallenges:

- B Team : Kevin Y. Yip, Roger P. Alexander, Koon-Kiu Yan, and Mark Gerstein of Yale University, USA

**Table 8:** *P*-values for the area under the ROC in the *in silico* size 100 network inference challenge.

	Ecoli1	Ecoli2	Yeast1	Yeast2	Yeast3	Summary
Team	*p*-value	*p*-value	*p*-value	*p*-value	*p*-value	*p*-value
B Team	1 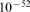	6 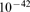	4 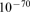	6 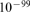	2 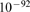	3 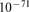
Bonneau	1 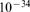	5 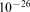	1 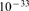	6 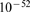	9 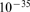	3 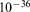
IntigernHSP	3 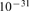	2 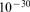	3 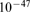	3 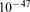	6 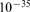	3 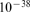
Team 305	1 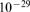	4 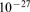	8 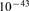	7 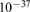	2 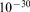	2 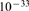
Team 301	1 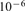	3 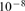	4 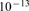	4 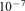	9 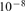	1 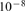
Team 310	8 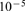	1 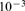	5 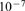	3 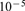	1 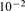	1 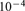
Team 314	4 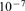	2 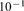	5 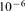	7 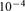	1 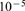	8 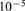
Team 183	4 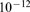	3 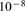	3 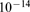	1 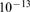	9 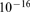	8 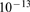
Team 254	4 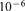	3 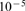	5 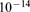	5 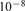	3 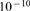	1 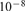
Team 192	3 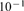	2 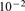	1 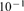	1 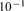	2 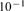	1 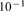
Team 110	8 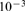	3 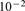	2 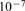	2 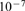	3 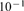	3 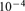
Team 303	5 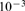	1 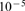	5 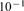	1 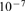	1	1 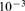
Team 236	1 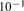	4 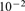	8 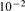	3 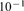	3 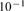	1 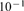
Team 283	6 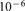	2 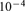	3 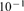	4 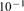	4 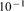	9 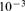
Team 291	2 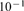	5 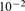	1	5 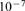	1	2 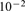
Team 271	3 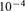	4 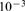	3 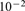	1 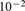	2 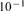	1 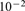
Team 273	2 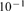	1 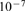	8 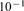	2 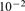	1 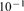	8 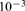
Team 70	4 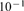	8 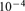	4 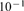	3 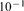	1 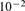	6 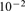
Team 302	2 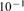	5 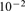	8 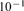	7 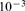	5 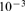	5 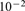
Team 269	2 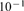	6 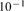	2 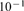	4 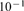	5 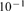	3 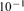
Team 282	6 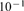	4 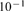	5 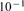	6 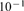	6 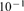	5 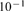
Team 280	6 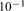	6 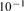	6 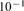	7 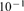	7 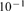	6 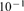

*P*-values for the area under the ROC curve for each of the five networks in the size-100 sub-challenge and a summary *p*-value (geometric mean) are indicated. The table is sorted in the same order as Table 9.

**Table 9:** Results of the *in silico* size 100 network inference challenge.

	AUROC	AUPR	Overal
Team	*p*-value	*p*-value	Score
B Team	3 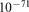	0	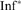
Bonneau	3 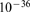	4 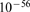	45.44
IntigernHSP	3 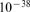	1 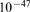	42.24
Team 305	2 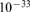	8 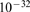	31.88
Team 301	1 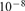	8 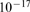	11.99
Team 310	1 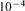	2 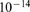	8.83
Team 314	8 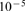	3 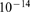	8.81
Team 183	8 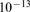	4 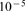	8.25
Team 254	1 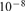	2 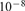	7.83
Team 192	1 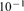	8 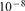	4.05
Team 110	3 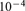	9 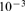	2.79
Team 303	1 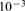	5 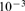	2.61
Team 236	1 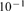	3 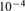	2.21
Team 283	9 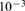	7 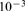	2.12
Team 291	2 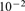	5 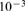	1.99
Team 271	1 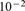	3 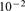	1.75
Team 273	8 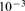	1 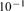	1.53
Team 70	6 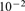	3 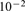	1.41
Team 302	5 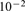	1 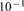	1.17
Team 269	3 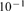	8 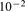	0.78
Team 282	5 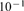	6 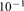	0.24
Team 280	6 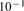	8 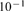	0.17

*The *p*-value for this performance was below the precision of our calculation.

Runners-up were identified by scores that were orders of magnitude more significant than the community at large, but not as significant as the best-performer:

- USMtec347 (10-node, 50-node): Peng Li and Chaoyang Zhang of the University of Southern Mississippi, USA
- Bonneau (100-node): Aviv Madar, Alex Greenfield, Eric Vanden-Eijnden, and Richard Bonneau of New York University, USA
- Intigern HSP (100-node): Xuebing Wu, Feng Zeng, and Rui Jiang of Tsinghua University, China

The overall *p*-values for the 100-node subchallenge (Table 9) demonstrates that the best teams predicted significantly better than the null model—a randomly sorted prediction list. However, the majority of teams did not predict much better than the null model. In the 10-node subchallenge, twenty-six of twenty-nine teams did not make statistically significant predictions on the basis of the AUROC (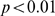). Fourteen of 27 teams in the 50-node subchallenge did not make significant predictions (AUROC 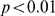). Eight of 22 teams in the 100-node subchallenge did not make significant predictions (AUROC 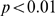). This is a sobering result for the efficacy of the network inference community. In Conclusions we discuss some reasons for this seemingly distressing result.

Some teams' methods were well-suited to smaller networks, others to larger networks (Table 10). This may have less to do with the number of nodes and more to do with the relative sparsity of the larger networks since the number of potential edges grows geometrically with the number of nodes (i.e., 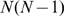).

**Table 10:** Comparison of scores in the 10, 50, and 100-node subchallenges.

*In Silico* Size 10			*In Silico* Size 50			*In Silico* Size 100		
Rank	Team	Score	Rank	Team	Score	Rank	Team	Score
1	**B Team**	5.12	1	**B Team**	39.83	1	**B Team**	Inf*
2	**USMtec347**	3.82	2	**USMtec347**	31.34	2	**Bonneau**	45.44
3	**IntigernHSP**	2.19	3	Team 256	17.93	3	**IntigernHSP**	42.24
4	Team 258	2.11	4	**Bonneau**	15.87	4	Team 305	31.88
5	**Bonneau**	2.07	5	**IntigernHSP**	13.77	5	Team 301	11.99
6	Team 305	1.94	6	Team 305	12.20	6	Team 310	8.83
7	Team 256	1.83	7	Team 258	8.40	7	Team 314	8.81
8	Team 110	1.13	8	Team 254	5.12	8	Team 183	8.25
9	Team 273	1.09	9	Team 310	4.84	9	Team 254	7.83
10	Team 183	1.06	10	Team 183	4.13	10	Team 192	4.05
11	Team 310	1.03	11	Team 301	3.56	11	Team 110	2.79
12	Team 282	1.02	12	Team 273	3.42	12	Team 303	2.61
13	Team 86	1.00	13	Team 314	3.15	13	Team 236	2.21
14	Team 303	0.97	14	Team 110	2.32	14	Team 283	2.12
15	Team 70	0.96	15	Team 192	1.90	15	**USMtec347**	1.99
16	Team 254	0.86	16	Team 303	1.83	16	Team 271	1.75
⋮	⋮	⋮	⋮	⋮	⋮	⋮	⋮	

*The *p*-value for this performance was below the precision of our calculation.

Best-performers are indicated in bold.

B Team used a collection of unsupervised methods to model both the genetic perturbation data (steady-state) and the dynamic trajectories [25]. Most notably, they correctly assumed an appropriate noise model (additive noise), and characterized changes in gene expression relative to the typical variance observed for each gene. It turned-out that this simple treatment of measurement noise was credited with their overall exemplary performance. This conclusion is based on our own ability to recapitulate their performance using a simple method that also uses a noise model to infer connections (see analysis of null-mutant *Z*-scores below). Additionally, B Team employed a few formulations of ODEs (linear functions, sigmoidal functions, etc.) to model the dynamic trajectories. In retrospect, their efforts to model the dynamic trajectories probably had a minor effect on their overall performance. Team Bonneau applied and extended a previously described algorithm, the Inferelator [26], which uses regression and variable selection to identify transcriptional influences on genes [27]. The methodologies of B Team and the other best-performers are described in separate publications in the PLoS ONE Collection.

#### A simple method: null-mutant *z*-score

We investigated the utility of a very simple network inference strategy which we call the null-mutant *z*-score. This strategy is a simplification of conditional correlation analysis [28]. Suppose there is a regulatory interaction which we denote A
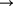
B. We assume that a large expression change in B occurs when A is deleted (compared to the wild-type expression). We compute the *z*-score for the regulatory interaction A
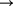
B
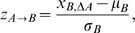
where 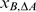 is the value of B in the strain in which A was deleted, 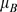 is the mean value of B in all strains (WT and mutants), and 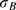 is the standard deviation of B in all strains. This calculation is performed for all directed pairs (A, B). We assume that 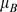 represents baseline expression (i.e., most gene deletions do not affect expression of B) and that deletion of direct regulators produces larger changes in expression than deletion of indirect regulators. Then, a network prediction is achieved by taking the absolute value of *z*-score and ranking potential edges from high to low values of this metric. Of note, the *z*-score prediction would have placed second, first, and first (tie) in the 10-node, 50-node, and 100-node subchallenges, respectively.

We do not imply that ranking edges by *z*-score is a superior algorithm for inferring gene regulation networks from null-mutant expression profiles in general, though conditional correlation has its merits. Rather, we interpret the efficacy of *z*-score for reverse-engineering these networks as a strong indication that an algorithm must begin with exploratory data analysis. Because additive Gaussian noise (i.e., simulated measurement noise) is a dominant feature of the data, *z*-score happens to be an efficacious method for discovering causal relationships between gene pairs. Furthermore, *z*-score can loosely be interpreted as a metric for the “information content” of a node deletion experiment. Subsequently, we will evoke this concept of information content to investigate why some network edges remain undiscovered by the entire community.

#### Intrinsic impediments to network inference

Analysis of the predictions of the community as a whole shed light on two important technical issues. First, are certain edges easy or difficult to predict and why? Second, do certain network features lead teams to predict edges where none exist? We call the former concept the *identifiability* of an edge, and we call the latter concept *systematic false positives*. A straightforward metric for quantifying identifiability and systematic false positives is the number of teams that predict an edge at a specified cutoff in the prediction lists. In the following analysis, we used a cutoff of 2P (i.e., twice the number of actual positives in the gold standard), which means that the first 2P edges were thresholded as present (positives). Incomplete prediction lists were completed with a random ordering of the missing potential edges prior to thresholding.

**Figure 6:**
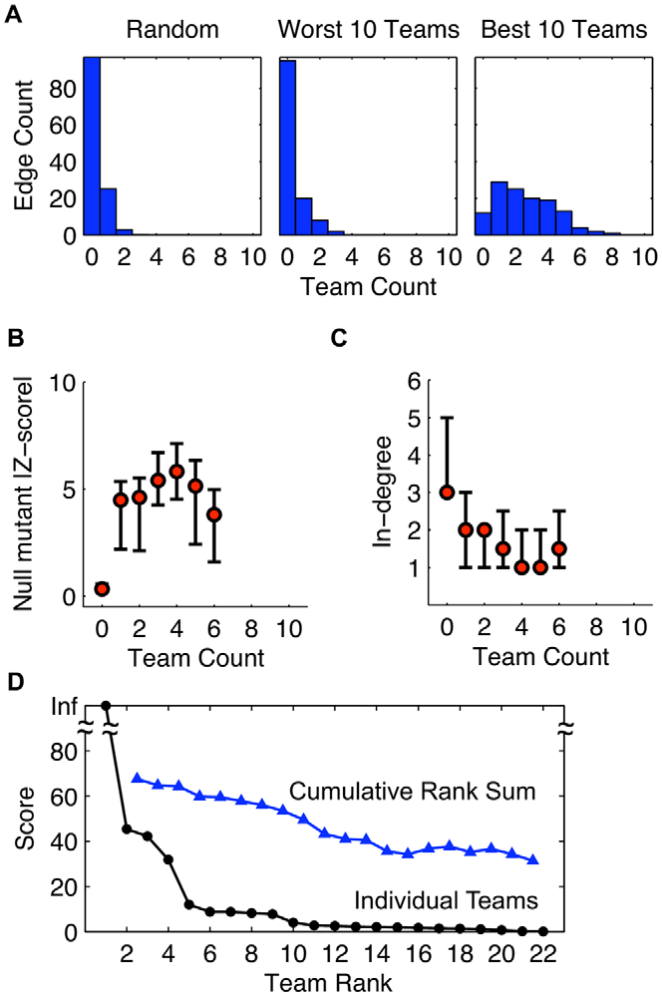
Analysis of the community of teams reveals characteristics of identifiable and unidentifiable network edges. The number of teams that identify an edge at a specified cutoff is a measure of how easy or difficult an edge is to identify. In this analysis we use a cutoff of 2P (i.e., twice the number of actual positive edges in the gold standard network). (**a**) Histograms indicate the number of teams that correctly identified the edges of the gold standard network called *InSilico_Size100_Ecoli1*. The ten worst teams in the 100-node sub-challenge identified about the same number of edges as is expected by chance. By contrast, the ten best teams identified more edges than is expected by chance and this sub-community has a markedly different *identifiability distribution* than random. Still, some edges were not identified by even the ten best teams (see bin corresponding to zero teams). Unidentified edges are characterized by (**b**) a property of the measurement data and (**c**) a topological property of the network. (**b**) Unidentified edges have a lower null-mutant absolute *z*-score than those that were identified by at least one of the ten best teams. This metric is a measure of the information content of the measurements. (**c**) Unidentified edges belong to target nodes with a higher in-degree than edges that were identified by at least one of the ten best teams. Circles denote the median and bars denote upper and lower quartiles. Statistics were not computed for bins containing less than four edges. (**d**) The benefits of combining the predictions of multiple teams into a consensus prediction are illustrated by the rank sum prediction (triangles). Though no rank sum prediction scored higher than the best-performer, a consensus of the predictions of the second and third place teams boosted the score of the second place team. Rank sum analysis shown for the 100-node sub-challenge.

We grouped the gold standard edges into bins according to the number of teams that identified the edge at the specified threshold (2P). We call the resulting histogram the identifiability distribution (Figure 6A). A community composed of the ten worst-performing teams has an identifiability distribution that is approximately equivalent to that of a community of random prediction lists—the two-sample Kolmogorov-Smirnov test *p*-value is 0.89. By contrast, a community composed of the ten best teams has a markedly different identifiability distribution compared to a random community—the two sample K-S test *p*-value is 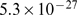.

The zero column in the identifiability distribution corresponds to the edges that were not identified by any team. We hypothesized that the unidentified edges could be due to a failure of the data to reveal the edge—the problem of insufficient information content of the data. Using the null-mutant *z*-score as a measure of the information content of the data supporting the existence of an edge, we show that unidentified edges tend to have much lower absolute *Z*-scores compared to the edges that were identified by at least one team (Figure 6B). This can occur if expression of the target node does not significantly change upon deletion of the regulator. For example, a target node that implements an OR-gate would be expected to have little change in expression upon the deletion of one or another of its regulators. Such a phenomena is more likely to occur for nodes that have a higher in-degree. Indeed, the unidentified edges have both lower *z*-score and higher target node in-degree than the identified edges (Figure 6C).

**Figure 7:**
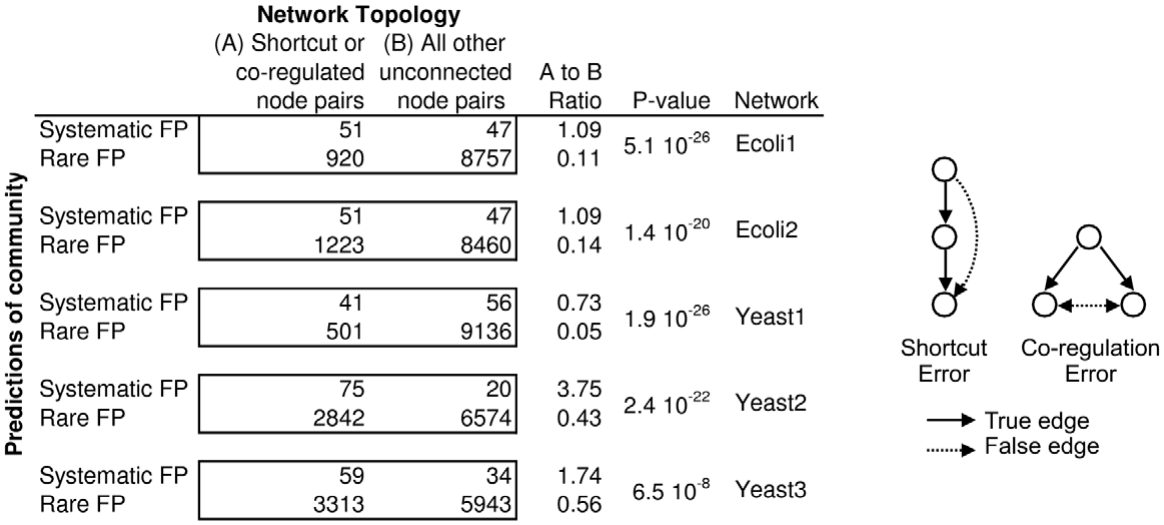
Community analysis of systematic false positives. Systematic false positive (FP) edges are the top one percent of edges that were predicted by the most teams to exist, yet are actually absent from the gold standard (i.e., negative). Rare false positive edges are the remaining 99 percent of edges that are absent from the gold standard network. The entries of each two-by-two contingency table sum to the total number of negative edges (i.e., those not present) in the gold standard network. There is a relative concentration of FP errors in the shortcut and co-regulated topologies, as evidenced by the A-to-B ratio. *P*-values for each contingency table were computed by Fisher's exact test, which expresses the probability that a random partitioning of the data will result in such a contingency table.

We investigated whether certain structural features of the gold standard networks led the community to incorrectly predict edges where there should be none. When multiple teams make the same false positive error, we call it a systematic false positive. The number of teams that make the error is a measure of confusion of the community. An ever-present conundrum in network inference is how to discriminate direct regulation from indirect regulation. We hypothesized that two types of topological properties of networks could be inherently confusing, leading to systematic false positives. The first type is what we call shortcut errors, where a false positive shortcuts a linear chain. A second type of direct/indirect confusion is what we call a co-regulation error, where co-regulated genes are incorrectly predicted to regulate one another (see schematic associated with Figure 7).

We performed a statistical test to determine if there is a relationship between systematic false positives and the shortcut and co-regulated topologies (Figure 7). Fisher's exact test is a test of association between two types of classifications. First, we classified all negatives (absence of edges) by network topology as either belonging to the class of shortcut and co-regulated node pairs, or not. Second, we classified negatives by the predictions of the community as either systematic false positives, or not. Finally, we constructed the 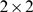 contingency table, which tabulates the number of negatives classified according to both criteria simultaneously.

There is a strong relationship between systematic false positives and the special topologies that we investigated. The systematic false positives are concentrated in the shortcut and co-regulated node pairs. This can be seen by inspection of each 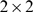 contingency table. For example, systematic false positives (the most common false positive errors in the community) have a ratio of 1.09 (51 special topologies to 47 generic topologies) whereas the less common false positive errors have a ratio of 0.11 (920 special topologies to 8757 generic topologies)—a profound difference in the topological distribution of false positives depending on whether many teams or few (including none) made the error. Direct-indirect confusion of this kind explains about half of the systematic false positives in the Ecoli1 network, and more than half in the other 100-node networks.

#### Community intelligence

Does the community possess an intelligence that trumps the efforts of any single team? To test this hypothesis we experimented with various ways of combining the predictions of multiple teams into a consensus prediction. Based simplicity and performance, we settled on the rank sum. The order of the edges in a prediction list is a ranking. We summed the ranks for each edge given by the various teams, then re-ranked the list to produce the consensus network prediction. Depending on which teams are included, this procedure can boost the overall score. For example, combining the predictions of the second and third-place teams achieved a better score than the second place team (Figure 6D). This result seems to indicate that the predictions of second and third-place teams are complementary; probably these teams took advantage of different features in the data. However, combining predictions with those of the best-performer only degraded the best score. Obviously, if the best prediction is close to optimal, combination with a suboptimal prediction degrades the score.

Starting with the second place team and including progressively more teams, the rank sum prediction score degrades much slower than the score of the individual teams (Figure 6D). This is reassuring since, in general, given the output of a large number of algorithms, we may not know which algorithms have efficacy. The rank sum consensus prediction is robust to the inclusion of random prediction lists (the worst-performing teams predictions were equivalent to random). It seems to be efficacious to blend the results of a variety of algorithms that approach the problem from different perspectives. We expect hybrid strategies to become more common in future DREAM challenges.

#### Lessons for experimental validation of inferred networks

This challenge called for the submission of a ranked list of predicted edges from most confidence to least confidence that an edge is present in the gold standard. Ranked lists are common for reporting the results of high-throughput screens, whether experimental (e.g., differential gene expression, protein-protein interactions, etc.) or computational. In the case of computational predictions, it is typical to experimentally validate a handful of the most reliable predictions. This amounts to characterizing the precision at the top of the prediction list. The outcome of the *in silico* network inference challenge reveals two reasons why a “top ten” approach to experimental validations is difficult to interpret.

Experimental validations of the handful of top predictions of an algorithm would be useful if precision were a monotonically decreasing function of the depth 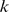 of the prediction list. The actual P-R curves illustrate that this is not the case. In Figure 5A, the best-performer initially had low precision which rose to a high value and was maintained to a great depth in the prediction list. The second-best-performer initially had high precision, which plummeted abruptly with increasing 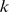. Validation of the top ten predictions would have been overly pessimistic in the former case, and overly optimistic in the latter case. Unfortunately, since precision is not necessarily a monotonically decreasing function of 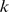, a small number of experimental validations at the top of the prediction list can not be extrapolated.

#### Year-over-year comparison

We would like to know if predictions are getting more accurate from year to year, and if teams are improving. With only two years of data available, no definitive statement can be made. However, there is one interesting observation from the comparison of individual teams' year-over-year scores. We compared the results of the 50-node subchallenge of DREAM3 to the results of the 50-node subchallenge of DREAM2 (the subchallenge that was substantially similar from year to year). It is a curious fact that teams that scored high in DREAM2 did not score high in DREAM3. There can be many reasons for the counter-trend. The *in silico* data sets were generated by different people from year to year. Furthermore, the topological characteristics of the networks were different. For example, all of the DREAM3 networks were devoid of cycles whereas the DREAM2 networks contained more than a few. The dynamics were implemented using different, though qualitatively similar equations. Finally, the current year data included additive Gaussian noise, whereas the prior data sets did not. Given the efficacy of directly acknowledging the measurement noise in the reverse engineering algorithm (e.g., null mutant *z*-score described above), any team that did not acknowledge the noise would have missed an important aspect of the data. We interpret the year-over-year performance as an indication that no algorithm is “one-size-fits-all.” The *in silico* network challenge data was sufficiently unique from year to year to warrant a custom solution. A final note, teams may have changed their algorithms.

#### Survey of methods

**Figure 8:**
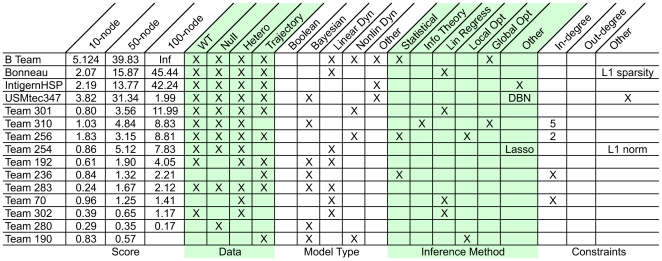
Survey of *in silico* network methods. There does not seem to be a correlation between methods and scores, implying that success is more related to the details of implementation than the choice of general methodology.

A voluntary survey was conducted at the conclusion of DREAM3 in which 15 teams provided basic information about the class of methods used to solve the challenge (Figure 8). The two most common modeling formalisms were Bayesian and linear/nonlinear dynamical models, which were equally popular (7 teams). Linear regression was the most popular data fitting/inference technique (4 teams); statistical (e.g., correlation) and local optimization (e.g., gradient descent) were the next most popular (2 teams). Teams that scored high tended to enforce additional constraints, such as minimization of the L1 norm (i.e., a sparsity constraint). Also, high-scoring teams did not ignore the null-mutant data set. The main conclusion from the survey of methods is that there does not seem to be a correlation between methods and scores, implying that success is more related to the details of implementation than the choice of general methodology.

## Conclusions

A macro-level goal of the DREAM project is to discover new biological knowledge from the aggregate efforts of the challenge participants. So far, we have not realized this lofty goal although we believe it will be possible for new knowledge to emerge from future DREAM challenges. This will require that teams build models that are simultaneously predictive and interpretable. Currently, the models offered in response to the DREAM challenges seem to be one or the other, but not both simultaneously. This is reasonable since the DREAM3 challenges solicited either measurement predictions or network predictions, but not both. Some of the DREAM4 challenges, which as of this writing are underway, attempt to remedy this disconnect.

Predicting measurements falls within the classic statistical learning paradigm whereby a training set is used to learn a model and a test set is used to evaluate how well the model generalizes. Regression-type methods performed well in this type of challenge. By comparison, biological network inference is less of a proven science. Perhaps it is this “wild west” character that attracts such high levels of participation in the DREAM challenges. The best-performer in the *in silico* network inference challenge appropriately handled measurement noise after exploring the character of the data. *Ad hoc* procedures based on exploratory data analysis seem to be rewarded by the *in silico* network inference challenge.

### Lessons Learned from Network Inference

After pouring over the predictions of the systems biology modeling community we have learned one overriding lesson about modeling and prediction of intracellular networks. There is no such thing as a one-size-fits-all algorithm. An algorithm has no intrinsic value in isolation of the data that motivated its creation. DREAM identifies the best teams with respect to specific challenges, not the best algorithms. This is an important distinction to keep in mind when interpreting results, especially results of the *in silico* challenge where the data is admittedly non-biological despite our best efforts. The matching of algorithm to data is fundamental for efficacy.

It would be inappropriate to dismiss an algorithm on the basis of a lackluster DREAM score. As a sanity check, we ran a well-respected network inference algorithm on the *in silico* data set. We do not name the algorithm or its authors, in keeping with one of the founding principles of DREAM—do no harm. Surprisingly, the algorithm, which is described or applied in string of high-profile publications, did not make statistically significant network predictions. Upon further examination of the data, we realized that the signal required by this particular algorithm was nearly absent from the *in silico* data set.

The perturbations used in the *in silico* data set are inappropriate for methods that expect pairs of linked nodes to covary under many conditions (e.g., correlation-based methods). In this data set, parent node mutations resulted in large expression changes in their direct targets. However, small expression changes due to indirect effects were not prominent. This is why null-mutant *z*-score was an efficacious signal for network inference, but measures of statistical dependence were not.

The Boolean-like character of the data was probably a consequence of the fact that there were no feedback loops in any of the fifteen *in silico* networks, which were extracted as subgraphs of the known *E. coli* and *S. cerevisiae* gene regulation networks. Although it is true that explicit feedbacks are exceedingly rare in the transcriptional networks of these organisms, there is extensive feedback control exerted at the level of protein-protein and protein-metabolite interactions, which was not represented in the generative model. Nevertheless, the generative model used in this challenge may provide a qualitatively accurate depiction of unicellular gene expression.

Since parent-child correlations are present in certain real-world gene expression data sets where correlation-based algorithms have been successfully applied, we cannot conclude that measures of statistical dependence are a poor choice for reverse-engineering gene networks in general, only for this particular data set. The *in silico* challenge seems to reward strategies that are guided by exploratory analysis of the data itself and that are adapted to the perturbations that exercise the network, and presumably penalizes established algorithms which may be based on different characteristics of certain real data sets.

Another take-home lesson from DREAM3 is that top-ten style validations are nearly impossible to interpret because precision is rarely a monotonically decreasing function of the threshold 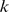. The best-performer and runner-up in the 100-node subchallenge illustrate the problem (see P-R curve in Figure 5B). The best-performer was identified because the overall network reconstruction was highly significant despite that the “high confidence” edge predictions were actually quite poor. By contrast, the runner-up in the 100-node subchallenge had very high precision for the high-confidence edge predictions but the overall network reconstruction was many orders of magnitude less significant than that of the best-performer. If the goal is to make a handful of predictions which are to be investigated by follow-up experiments, the latter situation is clearly desirable. If the goal is to sketch a large-scale network, the former situation is best. Both results are potentially desirable depending on the question at hand. However, in either case, validation of the top predictions gives no information about the overall trajectory of precision deeper into the edge list, and thus no information about the overall quality of the reconstructed network. Related to this last point, it may be that the best course of action is to combine the predictions of algorithms that focus on different aspects of the data into a consensus prediction, for example, by summing ranks and then re-ranking as we have illustrated in Figures 4B and 6D.

### Lessons Learned from Prediction of Measurements

The signaling response and gene expression prediction challenges were presented in typical machine learning style (e.g., training set/prediction set). Teams that adopted and embellished standard methods in machine learning such as the lasso (a variant of subspace regression) and k-nearest neighbors (a type of local estimation) predicted protein and mRNA expression well. It is interesting to ponder if prediction of measurements (from other measurements) may be relevant to experimental design of high-throughput molecular profiling studies.

We desire that models are interpretable in terms of plausible biology. However, extensive knowledge of signal transduction pathways were intentionally ignored by the best-performers in the signaling response prediction challenge. This turned-out to be a winning strategy, speaking to the power of statistical methods for prediction. Likewise, one of the best-performers in the gene expression prediction challenge ignored the known *S. cerevisiae* gene regulation network, but the other best-performer took advantage of it.

As evaluators, we do not have access to the models, only the predictions. Unfortunately, the predictions do not seem to deliver on our overarching goal of learning about the biology of signal transduction or gene expression. Even if we had access to the models, there is a dearth of interpretable biology to be learned from nearest-neighbor and regression approaches. In future runs of DREAM, we will strive to enhance the interpretability of the community's prediction efforts by incentivizing interpretability of models in terms of plausible biology. We believe that this community will rise to the challenge. It is interesting to ponder whether biologically plausible/interpretable models are at a disadvantage compared to regression-like methods when it comes to predicting measurements.

### Caution and Optimism

The vast majority of the teams' predictions were statistically equivalent to random guesses. It is likely that some of these teams employed methods that have previously been published and experimentally validated. This does not trouble us, since every algorithm is born from exploratory data analysis, so off-the-shelf applications of published algorithms would not be expected to perform well on fresh data sets. This has implications for the marketing of algorithms as being fit for a specific purpose, like gene regulation network inference. Even for this particular problem there is no one-size-fits-all algorithm. If algorithms *are* to be applied off-the-shelf, a strategy for guarding against bogus predictions is to employ a wide variety of algorithms that train on different features of the data (e.g., correlation [28], mutual information [29]–[32], synergy [33], etc.), then merge the results to gain confidence in the predictions.

The ill-posed signaling cascade identification challenge drew some fair criticism from participants who felt misled. One participant commented,

For me, the take-home message is that if you want to build a mathematical model to explain a dataset, you should have a good understanding of the dataset. In other words, modelers and experimentalists need to collaborate closely. I think that's the main problematic difference between DREAM and CASP. For [protein] structure determination the experimental and theory sides are relatively separable, but for network analysis the set of questions you can ask is so broad that the two sides need to work together to figure out both what experiments to do and how to analyze them afterwards.

The failure in execution of this challenge was due to a communication breakdown between the experimentalist who provided the data and the DREAM organizers. Had we, the organizers, been more experienced in the technical details of immunolabeling, the challenge would have written unambiguously. To the data producer, there was no ambiguity.

As challenge designers, we desire models that are simultaneously predictive and interpretable. Future runs of DREAM will encourage simultaneous submissions of networks and predictions, which may help us close-in on the macro-level goal of DREAM, to learn new biology from the aggregate predictions of the systems biology modeling community.

The exemplary predictions of the best-performers in each challenge are cause for celebration. As mentioned above, DREAM identifies the best teams, not the best algorithms. The importance of exploratory data analysis can not be stressed enough. The best-performers are exceedingly talented at their craft.
