# Correction: Towards a Rigorous Assessment of Systems Biology Models: The DREAM3 Challenges

**DOI:** 10.1371/annotation/f633213a-dc4f-4bee-b6c5-72d50e7073b8

**Published:** 2010-03-09

**Authors:** Robert J. Prill, Daniel Marbach, Julio Saez-Rodriguez, Peter K. Sorger, Leonidas G. Alexopoulos, Xiaowei Xue, Neil D. Clarke, Gregoire Altan-Bonnet, Gustavo Stolovitzky

In the Funding section a digit was missing from one of the grant numbers. NIH grant U54-CA11296 should read U54-CA112967. 

